# Interferon Regulatory Factors 1 and 2 Play Different Roles in MHC II Expression Mediated by CIITA in Grass Carp, *Ctenopharyngodon idella*

**DOI:** 10.3389/fimmu.2019.01106

**Published:** 2019-05-22

**Authors:** Xiao-Bing Lu, Zhao-Xi Wang, Shu-Bo Liu, Xiang-Yang Zhang, Long-Feng Lu, Shun Li, Dan-Dan Chen, Pin Nie, Yong-An Zhang

**Affiliations:** ^1^Chinese Academy of Sciences, Institute of Hydrobiology, Wuhan, China; ^2^College of Modern Agriculture Sciences, University of Chinese Academy of Sciences, Beijing, China; ^3^State Key Laboratory of Agricultural Microbiology, College of Fisheries, Huazhong Agricultural University, Wuhan, China; ^4^Laboratory for Marine Biology and Biotechnology, Qingdao National Laboratory for Marine Science and Technology, Qingdao, China

**Keywords:** CIITA, MHC II, IRF1, IRF2, IFN-γ, grass carp

## Abstract

Expression of major histocompatibility complex class II (MHC II) molecules, which determines both the immune repertoire during development and subsequent triggering of immune responses, is always under the control of a unique (MHC class II) transactivator, CIITA. The IFN-γ-inducible MHC II expression has been extensively and thoroughly studied in humans, but not in bony fish. In this study, the characterization of CIITA was identified and its functional domains were analyzed in grass carp. The absence of GAS and E-box in the promoter region of grass carp CIITA, might imply that the cooperative interaction between STAT1 and USF1 to active the CIITA expression, found in mammals, is not present in bony fish. After the transfection of IFN-γ or IFN-γ rel, only IFN-γ could induce MHC II expression mediated by CIITA. Moreover, interferon regulatory factor (IRF) 2, which cooperates with IRF1 to active the CIITA promoter IV expression in mammals, played an antagonistic role to IRF1 in the activation of grass carp CIITA. These data suggested that grass carp, compared with mammals, has both conservative and unique mechanisms in the regulation of MHC II expression.

## Introduction

In vertebrates, major histocompatibility complex II (MHC II) molecules play a pivotal role in the induction of immune responses by presenting exogenous antigenic peptides to CD4^+^ helper T lymphocytes, resulting in their activation and differentiation (1). MHC II molecules are also crucial for the maintenance of self-tolerance, in which the survived T cells carrying TCR can recognize self-MHC molecules (2). Although MHC II molecules are constitutively expressed in professional antigen-presenting cells (APCs), such as B cells, dendritic cells (DCs), and macrophages (3), other non-APCs including mesenchymal stromal cells, fibroblasts, and endothelial cells, can also be induced to express MHC II after stimulation with interferon (IFN)-γ (4, 5). Both constitutive and induced MHC II expression can be further modulated by additional signals. For instance, the constitutive MHC II expression is regulated as a function of the developmental stage and can be modulated by various cytokines in B cells and DCs. IFN-γ-induced expression of MHC II can be inhibited by numerous stimuli such as TGF-β and IL-10 (6).

The regulation of MHC II genes occurs primarily at the transcriptional level (7). The MHC class II transactivator (CIITA), is a coactivator that regulates MHC II transcription (8), interacting with numerous DNA binding factors in the cell nucleus, including nuclear factor Y (NFY), regulatory factor X (RFX), and cyclic AMP response element binding protein (CREB) (9, 10). These interactions mainly depend on distinct structural domains within CIITA. The CIITA has a complex domain structure which contains an N-terminal acidic domain (NAD), proline-, serine-, and threonine-rich (PST) regions, GTP-binding domains (GBDs), at least one nuclear localization sequence (NLS), and a series of leucine-rich repeats (LRRs). RFX and NFY bind to the NAD, and the other transactivators interact with the GBDs. This active complex, or enhanceosome, binds to the conserved regulatory elements of the MHC II promoter and then turn on the expression of MHC II. Interestingly, CIITA does not directly bind any of the regulatory elements that are involved in MHC II expression in this process, but only acts as a transcriptional coactivator via protein-protein interactions with other DNA-binding proteins that bind to the CIITA promoter (11).

In mammals, CIITA is controlled by multiple promoters, leading to diverse CIITA transcripts with different first exons. Interestingly, these promoters are activated in a selective manner. Promoter I and promoter III, respectively manages specific constitutive expression in DCs and B cells, whereas promoter IV mediates IFN-γ inducible expression in most MHC II-negative IFN-γ inducible cells (12). Promoter IV of CIITA, which has numerous cis-acting elements including an IFN-γ activation sequence (GAS, TTCNNNNGAA) and E-box (CACGTG), as well as an interferon stimulation response element (ISRE), is activated in response to the classical IFN-γ-mediated signaling pathway (13, 14). Binding of IFN-γ to its receptor induces the activation of Janus kinase (JAK) 1 and JAK2, which leads to the phosphorylation, dimerization, and nuclear translocation of signal transducer and activator of transcription 1 (STAT1). Phosphorylated STAT1 can bind to the promoter of the downstream gene, such as interferon regulatory factor (IRF) 1, to activate its expression (15). By binding to the ISRE motif of CIITA promoter IV, IRF1 could induce CIITA expression (16).

In teleost species, only the CIITA gene of channel catfish (*Ictalurus punctatus*) has been identified (17), and there are few reports about the study of the antigen presentation signaling pathway mediated by IFN-γ. Unlike mammals, the type II IFN system, containing IFN-γ and IFN-γ related (IFN-γ rel) genes, has been widely investigated in bony fish (18–22). In addition, the IRF1 subfamily, which regulates the expression of CIITA, is an important group with two IRF members in mammals: IRF1 and IRF2, while there is a third member named IRF11 in fish (23, 24). Therefore, it is necessary to explore the differences of the antigen presentation signaling pathway between mammals and fish and to uncover the roles of the involved molecules. To achieve this objective, grass carp (*Ctenopharyngodon idella*), one of the important economic aquaculture species, was chosen as the object of study. In this study, the CIITA, IFN-γ, IFN-γ rel, IRF1, IRF2, and IRF11 genes of grass carp were cloned and characterized, and their roles were researched in the antigen presentation signaling pathway.

## Materials and Methods

### Fish Treatment and Ethics Statement

Grass carp (12~15 cm in body length) obtained from Duofu fish farm (Wuhan, China) were cultured at recirculating aquaculture system for at least 2 weeks before tissue collection. All operations of fish were approved by the Committee on the Ethics of Animal Experiments of the Institute of Hydrobiology, Chinese Academy of Sciences.

### Cell Lines and Strains

*Ctenopharyngodon idella* kidney (CIK) cells and grass carp ovary (GCO) cells were cultured at 28°C in medium 199 including 10% inactivated fetal bovine serum (FBS, Invitrogen). Human 293T cells were maintained at 37°C in Dulbecco's modified Eagle's medium (DMEM) supplemented with 10% FBS. All cells were maintained in the atmosphere of 5% CO_2_. *Escherichia coli* (*E. coli*) DH5α was used for clone identification and plasmid amplification, and *E. coli* BL21(DE3) pLysS (Stratagene) was used for prokaryotic expression.

### Gene Cloning and Plasmid Construction

To clone the cDNA sequence of CIITA, degenerate primers (Table S1) were designed based on the conserved regions in CIITA cDNAs of other fish species, including *Danio rerio* (accession no. XM_009299437), *Cynoglossus semilaevis* (XM_008330114), *Stegastes partitus* (XM_008291843), and *Poecilia reticulata* (XM_008415765). PCR reactions were performed in 50 μl volumes with the template cDNA derived from spleen tissue and GCO cell lines. The PCR products were inserted into pMD-18T easy vector (Takara), transformed into *E. coli* DH5α, and then positive clones were randomly selected for sequencing.

For 5′-rapid amplification of cDNA end (RACE), cDNA was tailed with dCTP and terminal deoxynucleotidyl transferase (Promega), which was then used as template for the PCR with gene-specific reverse primers (Table S1). For 3′-RACE, forward primers (Table S1) were designed based on the above obtained cDNA fragments and used for the primary and nested PCRs. The PCR products were cloned and sequenced as described above. Full length cDNA sequence was confirmed by sequencing the PCR product amplified with the primers (Table S1) located at the 5′ and 3′ untranslated regions (UTRs).

The open reading frames (ORFs) of IFN-γ (JX657683), IFN-γ rel (FJ695519), IRF11 (MH797556), and CIITA of grass carp were respectively cloned into the *Kpn*I and *Xho*I sites of pcDNA3.1 (+) vector (Invitrogen) for over-expression. For IRF1 (JX965183) and IRF2 (JX628585), their ORFs and two truncated mutants, were generated by PCR and cloned into the *EcoR*I and *Xho*I sites of pcDNA3.1(+) vector. For promoter activity analysis, the promoter fragment of grass carp CIITA gene was constructed on basis of pGL3-Basic vector (Promega). For chromatin immunoprecipitation (ChIP) assays, IRF1 and IRF2, respectively contained an N-terminal Myc-tag and HA-tag, were inserted into *EcoR*I and *Xho*I sites of pcDNA3.1(+) vector. All constructed plasmids were verified by sequencing (Tsingke Biological Technology).

### Cell Transient Transfection, Analysis of Gene Expression and Luciferase Activity Assay

GCO cells were seeded in 6-well or 24-well plate and transfected with indicated plasmids by X-tremeGENE HP DNA Transfection Reagent (Roche) according to the manufacturer's instruction. In order to analyze the downstream gene expression induced by IFN-γ, the cell samples were collected with Trizol reagent at 0, 24, 48, or 72 h post-transfection. For luciferase activity assay, the cell samples were collected after 24 h transfection and washed with phosphate buffer saline (PBS) for three times, then lysed with Lysis Buffer (Promega) for 15 min on mechanical horizontal rotator, and finally measured luciferase activity by luciferase assay system (Promega). The assays were carried out independently three times and the average results were used for statistical analysis.

### Recombinant Protein Purification and Polyclonal Antibody Development

Partial IRF1 (amino acid number from 113 to 289) sequence was inserted into the *EcoR*I and *Xho*I sites of PET23b vector and then transformed into *E. coli* DH5α. Plasmids were extracted from the verified positive clones and transformed into *E. coli* BL21(DE3) pLysS for prokaryotic expression. Bacteria were grown in LB medium at 37°C to an OD_600_ of 0.6~0.8, and then induced with 1 mM isopropyl-β-D-thiogalactopyranoside (IPTG) at 37°C for 4–5 h. Cells were collected and resuspended in 25 ml native lysis buffer (50 mM NaH_2_PO_4_, 300 mM NaCl, and 10 mM imidazole). Cell suspensions were mechanically disrupted using an ATS Nano Homogenize Machine (ATS Engineering Inc.) and then centrifuged at 12,000 *g* for 15 min to pellet the inclusion bodies. Inclusion bodies were solubilized with 25 ml denaturing lysis buffer (50 mM NaH_2_PO_4_, 300 mM NaCl, 10 mM imidazole, and 8 M urea) and then incubated with Ni-NTA Super flow resin (Qiagen) by rocking at 4°C overnight. The resin was washed with wash buffer 1 (50 mM NaH_2_PO_4_, 300 mM NaCl, 20 mM imidazole, and 8 M urea) and wash buffer 2 (50 mM NaH_2_PO_4_, 300 mM NaCl, 40 mM imidazole, and 8 M urea) in turn. The bound protein was eluted with elution buffer (50 mM NaH_2_PO_4_, 300 mM NaCl, 250 mM imidazole, and 8 M urea), then packed into a dialysis tube and finally the elution buffer was replaced by PBS. Concentration of the purified recombinant protein was determined by SDS-PAGE and Pierce™ BCA Protein Assay Kit (ThermoFisher Scientific). The antisera against grass carp IRF1 were obtained by immunizing white rabbit with the purified recombinant protein. The polyclonal antibody (pAb) was purified using Protein G Column (GE Healthcare) from the antisera. The specificity of the pAb was determined by Western blot.

### Western Blot

Protein samples were separated on SDS-PAGE and transferred to PVDF membrane (BioRad). The membrane was blocked in TBST buffer (25 mM Tris-HCl, 150 mM NaCl, 0.1% Tween 20, pH 7.5) containing 5% skimmed milk for 1 h at room temperature. After incubation with primary antibody (diluted in TBST buffer containing 1% skimmed milk), the membrane was washed three times with TBST Buffer and then incubated with secondary antibody (diluted in TBST buffer containing 1% skimmed milk). After washing three times, the membrane was stained with Immobilon TM Western Chemiluminescent HRP Substrate (Millipore) and examined using an Image Quant LAS 4000 system (GE Healthcare). The antibodies against grass carp IRF1 and β-actin (Cell Signaling Technology) were diluted at 1:1,000 and HRP-conjugated anti-rabbit IgG (ThermoFisher Scientific) was diluted at 1:5,000.

### RNA Extraction and Quantitative Real-Time PCR (qRT-PCR)

Total RNA of tissues or cells samples were extracted by Trizol reagent (Invitrogen) and reverse transcribed by GoScript reverse transcription system (Promega). The qRT-PCR mixture consisted of 1 μl cDNA template, 3.5 μl nuclease-free water, 5 μl SYBR Green PCR master mix (BioRad), and 0.25 μl of each forward and reverse primers (10 μM). Real-time PCR was conducted using the following program: 1 cycle of 5 min at 95°C, 42 cycles of 30 s at 95°C, 30 s at 60°C and 30 s at 72°C, followed by melting curve from 65° to 95°C to verify the amplification of a single product. Grass carp beta-actin (β*-actin*) was used as internal control. The relative fold changes were calculated by comparison to the corresponding controls using the comparative CT (2^−ΔΔ*Ct*^) method. All primers used for qRT-PCR are shown in Table S1.

### Isolation of Head Kidney Leukocytes (HKLs)

Grass carp leukocytes were collected from head kidney as previously reported (25). Briefly, fish head kidneys were removed aseptically and pressed through a 40-μm nylon mesh and suspended in 1,640 medium (Gibco) supplemented with 100 U/ml penicillin, 100 μg/ml streptomycin, and 25 U/ml heparin. The cell suspensions from head kidney were layered onto the 51/34% discontinuous Percoll (GE Healthcare) density gradient and centrifuged at 400 *g* for 30 min. The band of leukocytes lying at the interface was collected and the cells were washed three times with medium supplemented with 100 U/ml penicillin, 100 μg/ml streptomycin, and 10 U/ml heparin. The cells were resuspended in complete medium (1,640 medium supplemented with 100 U/ml penicillin, 100 μg/ml streptomycin, and 10% FBS), seeded into 24-well plate (1 ml cells/ well) and then incubated at 28°C for further treatment.

### Cell Stimulation With Polyinosinic: Polycytidylic Acid (Poly I:C) and LPS

CIK cells were cultivated in 6-well plate for 12 h and then transfected with 2 μg/ml poly I:C or immediately stimulated with 25 μg/ml LPS or added PBS as control. The concentrations chosen for each stimulant were deemed optimal from previous studies (26, 27). Cell samples were washed with PBS for three times and harvested at 0, 6, 12, 24, 48, and 72 h after stimulation. For Western blot, the samples were lysed in Lysis buffer containing protease inhibitors. As for the gene expression analysis, samples were harvested with Trizol reagent. Freshly prepared HKLs, seeded in 24-well plate (1 × 10^6^ cells/ well), were stimulated with 50 μg/ml Poly I:C (Sigma-Aldrich) or 25 μg/ml LPS (Sigma-Aldrich) or PBS as control for 3, 6, 12, 24, 48, and 72 h. The stimulants were diluted in complete medium before addition to the cells. The treatments were terminated by dissolving the cells in TRIzol reagent.

### Chromatin Immunoprecipitation (ChIP) Assay

For the ChIP assay, 293T cells were cultured in the 10-cm dish and transfected with indicated plasmids. After 24 h, 270 μl of 37% formaldehyde were added to 10 ml growth media for the crosslink of intracellular protein-DNA complexes. After incubating at room temperature for 10 min, all dishes were added with 0.125 M glycine to quench unreacted formaldehyde, and then the culture media mixed with formaldehyde and glycine were discarded. After washing twice with cold PBS, the cell samples were respectively scraped into microfuge tubes and centrifuged at 4°C for 5 min, and then lysed with 1 ml of SDS Lysis Buffer (Beyotime). One third of cell lysates were prepared for ultrasonication, and then centrifuged at 4°C for 10 min to remove insoluble material. 100 μl of sheared crosslinked chromatin were added into the tube containing 900 μl of Dilution Buffer (Sigma-Aldrich). 60 μl of Protein A + G Agarose (Beyotime) were prepared to preclear the chromatin to remove proteins or DNA that may bind nonspecifically to agarose. After centrifugation, 10 μl of the supernatant were removed as input and the remaining supernatant were incubated with anti-Myc-Agarose or anti-HA-Agarose overnight at 4°C. Immunoprecipitated Myc-tagged protein-DNA or HA-tagged protein-DNA complexes were washed with low salt immune complex wash buffer (0.1% SDS, 1% Tritons X-100, 2 mM EDTA, 20 mM Tris-HCl, 150 mM NaCl), high salt immune complex wash buffer (0.1% SDS, 1% Tritons X-100, 2 mM EDTA, 20 mM Tris-HCl, and 500 mM NaCl), LiCl immune complex wash buffer (0.25 M LiCl, 1% NP40, 1% sodium deoxycholate, 1 mM EDTA, 10 mM Tris-HCl) and TE buffer (10 mM Tris-HCl, 1 mM EDTA) respectively. After washing, 100 μl Elution Buffer (Omega) were added to each tube containing the Myc-tagged protein-DNA complex and then flicked to fully mix with beads. After incubation for 15 min, the supernatants were collected into new microfuge tubes and the beads were discarded. All new tubes (immunoprecipitates and input) were treated with 8 μl 5 M NaCl at 65°C for 4–5 h, 1 μl RNase A at 37°C for 30 min, and a solution containing 4 μl 0.5 M EDTA, 8 μl 1 M Tris-HCl and 1 μl proteinase K at 45°C for 2 h. The DNA was purified using a PCR purification kit (Omega). Purified DNA was analyzed by semi-quantitative PCR using specific primers (Table S1).

### Statistics Analysis

Statistical analysis was carried out by one-way analysis of variance (ANOVA) with the Dunnett's *post-hoc* test (SPSS Statistics, Version 20). All experiments were repeated at least three times. The *p* < 0.05 was considered statistically significant.

## Results

### Molecular Characterization of Grass Carp CIITA

**Figure 1:**
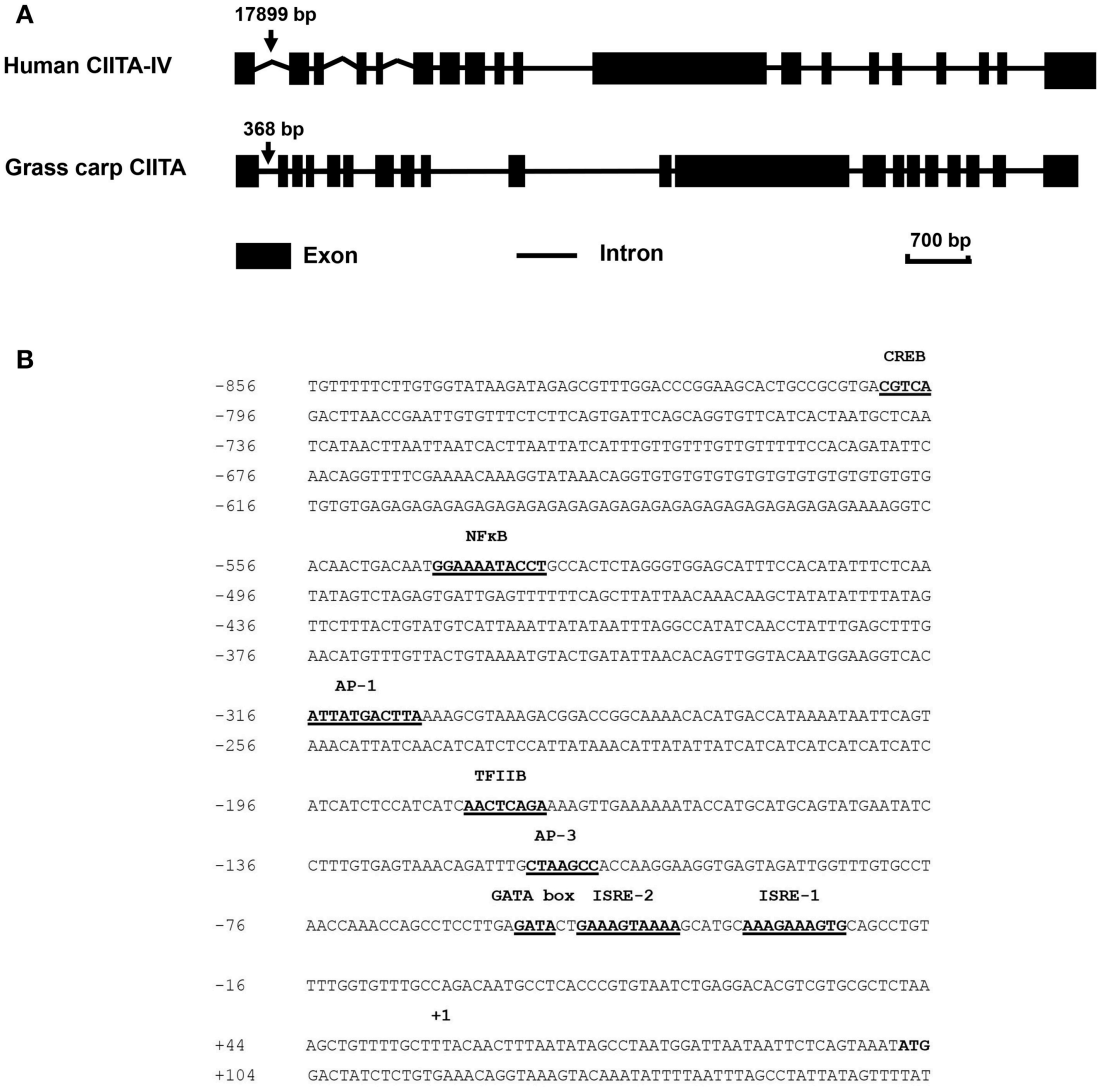
Schematic diagrams of the CIITA gene in human and grass carp, and prediction of the transcription factor binding sites in the promoter region of grass carp CIITA. **(A)** Human type IV CIITA gene sequence was obtained from UCSC Genome Browser, and grass carp CIITA gene sequence was acquired by local blast from grass carp genomic data downloaded from official National Center for Gene Research website (http://www.ncgr.ac.cn/grasscarp/). **(B)** The putative motifs including CREB, NFκB, AP-1, TFIIB, AP-3, GATA box, and two ISREs were predicated. The transcriptional start site was designated by +1.

**Figure 2:**
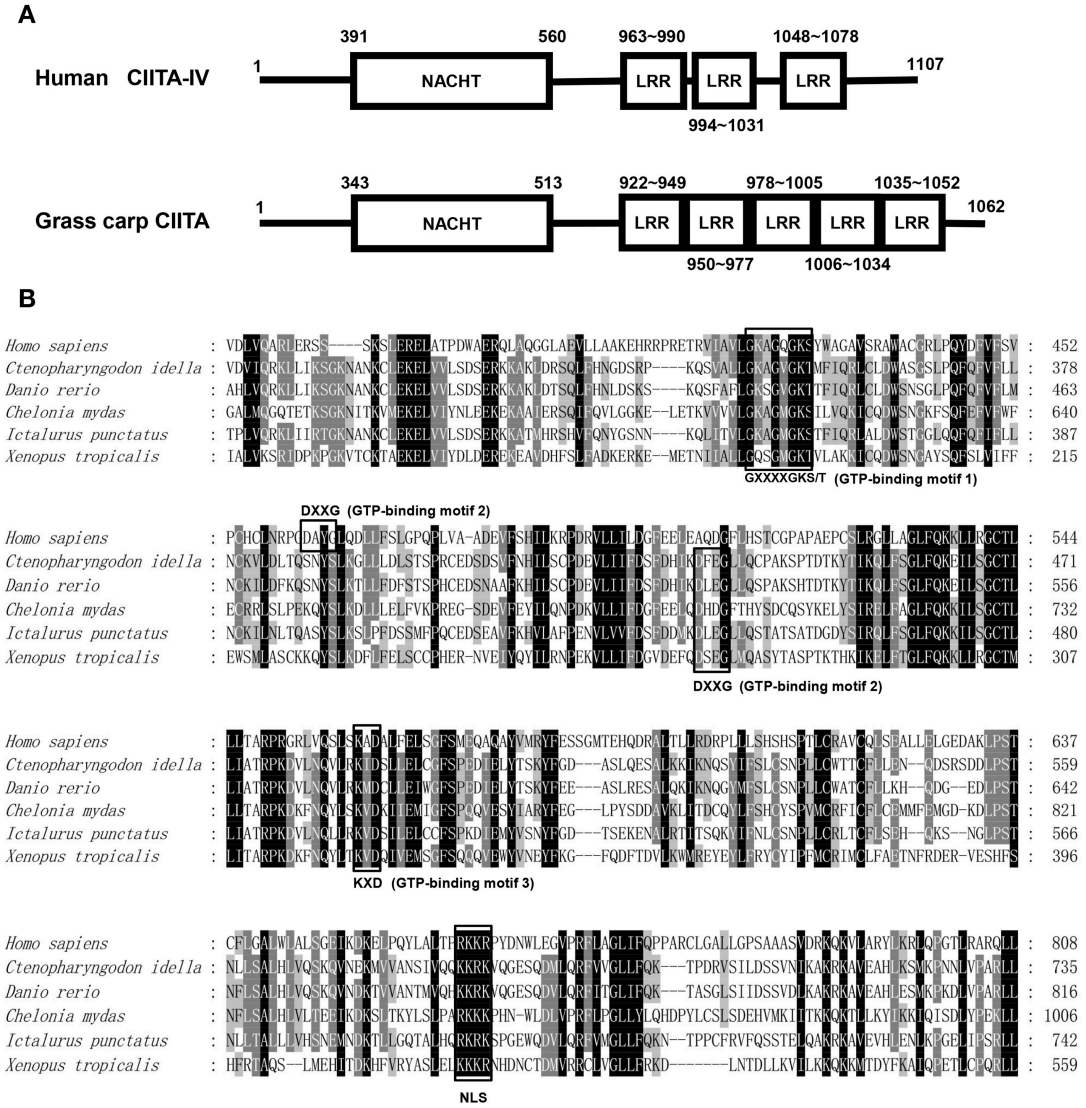
Protein sequence analysis of the CIITA. **(A)** NACHT domains and LLR domains of human and grass carp CIITA were identified with Smart BLAST in NCBI (https://www.ncbi.nlm.nih.gov/). **(B)** GTP-binding motifs and NLS were identified by the characteristic sequence analysis reported in human. The accession numbers of CIITA included in amino acid sequence alignment are as follows: *Homo sapiens*, NP_001273331; *Chelonia mydas*, XP_007058212; *Xenopus tropicalis*, XP_017952759; *Ictalurus punctatus*, AFL70283; *Danio rerio*, XP_009297712; *Ctenopharyngodon idella*, AXY05349.

The full-length cDNA sequence of grass carp CIITA was obtained by homologous cloning and RACE techniques and then was deposited in GenBank (accession number MH794281). It is 3,519 bp in length covering a 5′- UTR of 104 bp, an ORF of 3,189 bp, and a 3′-UTR of 226 bp with a polyadenylation signal (AATAAA) at 11 bp upstream of the poly (A) tail. A genomic structure analysis showed that grass carp CIITA gene contains 19 introns and 20 exons (Figure 1A), which is one more than the number of exons in human CIITA gene. Promoter sequence analysis showed that it includes 8 putative motifs, CREB, NFκB, AP-1, TFIIB, AP-3, one GATA box, and two ISREs (Figure 1B). Functional domain prediction showed that the putative CIITA protein contains a NACHT domain and five LRR domains, which is slightly different from that of humans (Figure 2A). CIITAs of six vertebrate species were chosen and aligned, and the result showed that grass carp CIITA shared high sequence homology in conserved domains (Figure 2B), including GTPase consensus motifs and NLS motifs. Phylogenetic analysis showed that grass carp CIITA was first clustered to zebrafish CIITA, then to those of other fish species (Figure S1).

### Tissue Expression of Grass Carp CIITA

To have an insight into the roles of grass carp CIITA, tissue distributions were examined by qRT-PCR (Figure S2). The expression profile of CIITA in skin was chosen as the cardinal, set as 1. CIITA displayed the highest expression level in head kidney (20.444-fold) and the lowest level in muscle (0.0747-fold) among the 10 test tissues.

### IFN-γ Induces MHC II Expression

**Figure 3:**
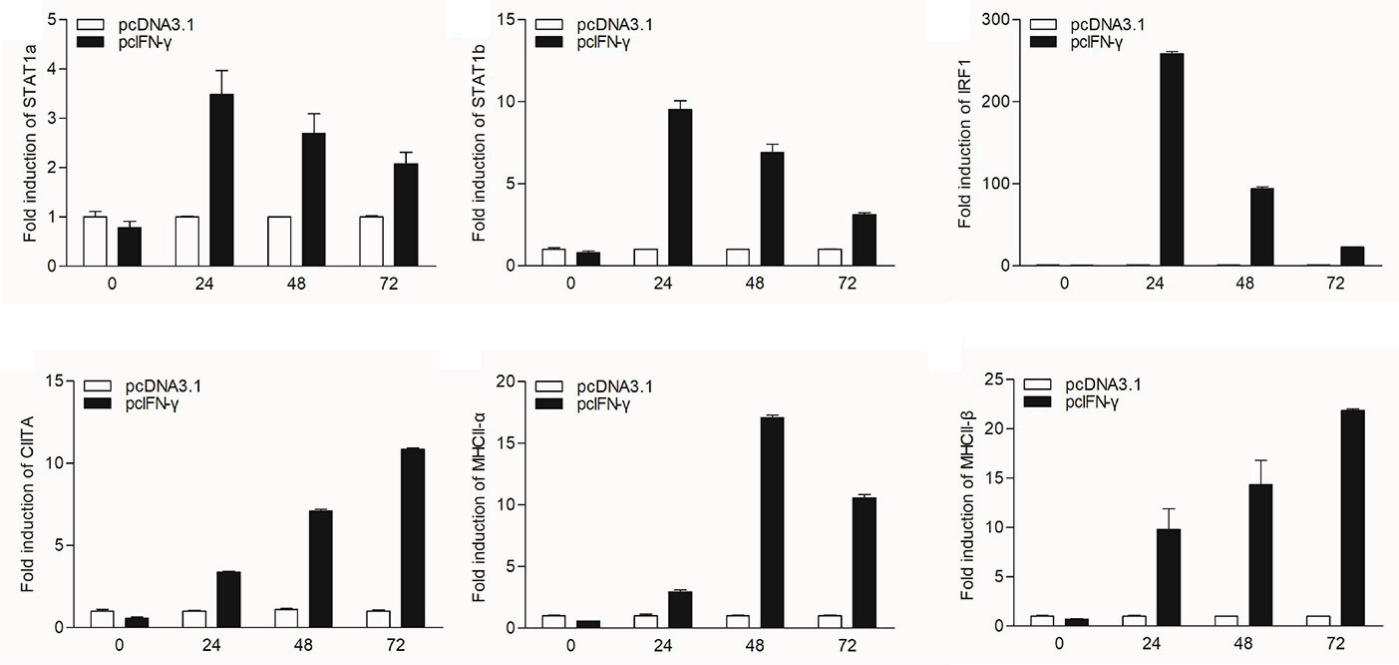
mRNA expression of the STAT1a, STAT1b, IRF1, CIITA, and MHC II *in vitro* triggered by IFN-γ. GCO cells were transfected with 1.5 μg pcDNA3.1 or pcIFN-γ respectively. Cells were harvested at 0, 24, 48 and 72 h after transfection. Then total RNAs were extracted to examine the mRNA levels of STAT1a, STAT1b, IRF1, CIITA, and MHC II through qRT-PCR.

In mammals, MHC II expression can be triggered by IFN-γ through STAT1 signaling pathway. To characterize the corresponding signaling pathway in grass carp, the eukaryotic expression plasmids of IFN-γ and IFN-γ rel were transfected into GCO cells. The qRT-PCR analysis indicated that IFN-γ, not IFN-γ rel, could activate IRF1 expression, and both IFN-γ and IFN-γ rel could not promote the expression of IRF2 and IRF11 (Figure S3). The expression of STAT1a, STAT1b, IRF1, CIITA, and MHC II was then detected at different time points: 0, 24, 48, and 72 h after IFN-γ transfection (Figure 3). STAT1a, STAT1b and IRF1 had high expression levels at 24 h, while high expression levels of CIITA and MHC II were at 48 and 72 h after transfection.

### IRF1 Promotes MHC II Expression Mediated by CIITA

**Figure 4:**
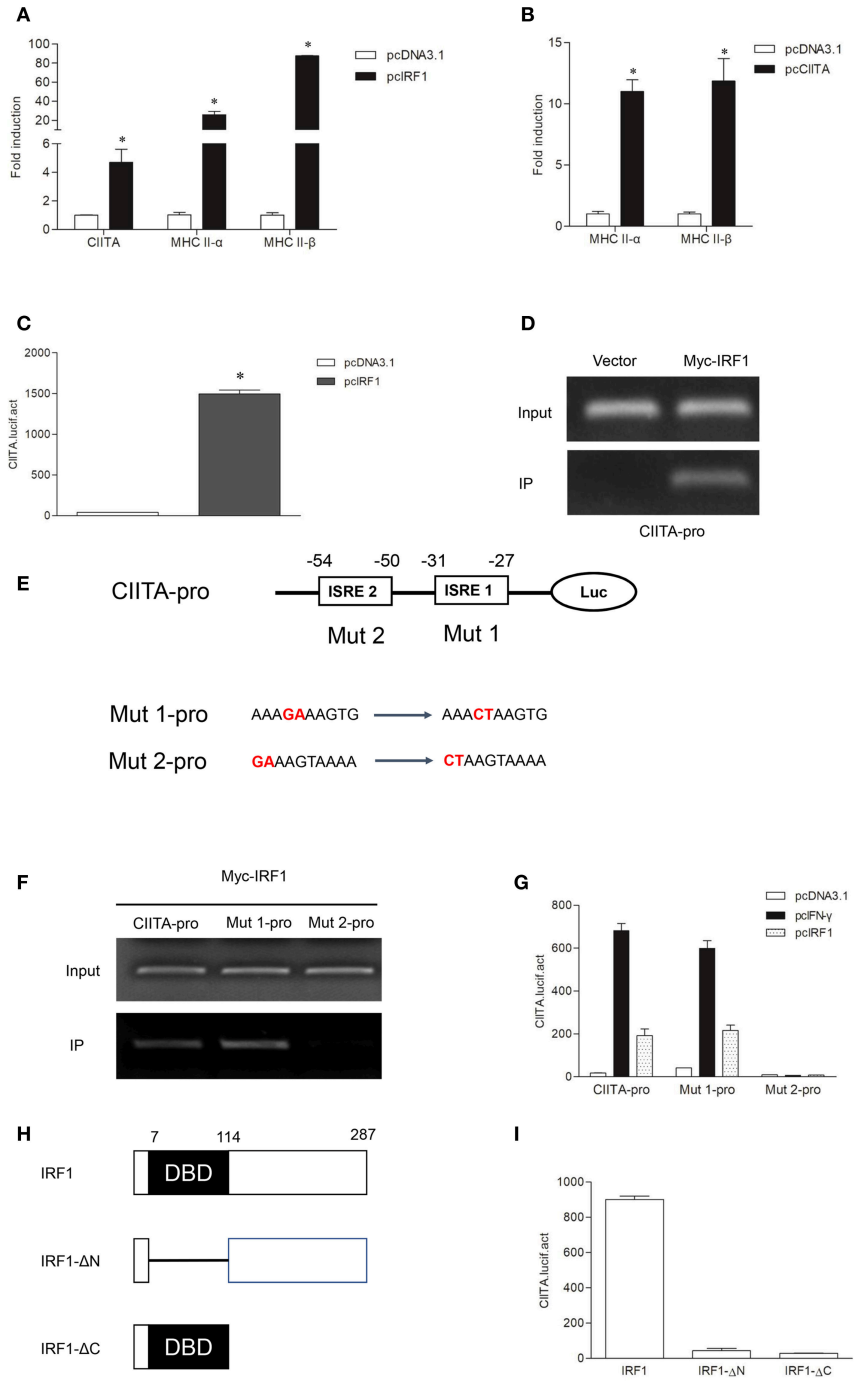
Expression of the MHC II gene activated by IRF1 and CIITA. **(A,B)** IRF1 and CIITA induce MHC II expression. GCO cells were transfected with 1.0 μg pcDNA3.1, pcIRF1, or pcCIITA respectively. Cells were harvested at 24 h after transfection and fold changes of the gene expression were measured through qRT-PCR. **(C)** Activation of grass carp CIITA promoter by IRF1. GCO cells seeded in 24-well plate were cotransfected with (0.25 μg pcDNA3.1 or pcIRF1) and CIITA promoter at the ratio of 1:1. pRL-TK was used as an internal control. Promoter activities were monitored at 24 h after transfection. Asterisks (^*^) indicate significant differences from control (*p* < 0.05). **(D)** The binding of IRF1 with CIITA promoter. 293T cells seeded in 10-cm dishes were cotransfected with 5 μg Myc-IRF1 and 5 μg CIITA-pro. Empty vector PCMV-Myc (5 μg) was transfected in parallel as control. After 24 h, cell lysates were immunoprecipitated with anti-Myc-Agarose beads. Then the DNA binding to CIITA promoter was checked by semiquantitative PCR. The input was used as a control in semiquantitative PCR to quantify the DNA concentration. **(E)** Schematic representation of mutated CIITA promoter-driving luciferase constructs. The base substitution mutations of each ISRE site are shown. **(F)** The binding of IRF1 with ISRE motifs of CIITA promoter. The cells seeded in 10-cm dishes were cotransfected with 5 μg Myc-IRF1 and (5 μg CIITA-pro, Mut 1-pro or Mut 2-pro). Cell samples were harvested at 24 h after transfection and cell lysates were immunoprecipitated with anti-Myc-Agarose beads. The input was used as a control in semiquantitative PCR to quantify the DNA concentration. **(G)** The function of ISRE motifs on activation of CIITA. GCO cells were cotransfected with (pcDNA3.1, IFN-γ, or IRF1) and (CIITA-pro, Mut 1-pro, or Mut 2-pro). Luciferase activities were monitored at 24 h after transfection. **(H)** Schematic representation of wild type IRF1 and its two mutants. **(I)** Effect of the two structural domains of IRF1 on the CIITA promoter. GCO cells were cotransfected with (IRF1, IRF1-ΔN or IRF1-ΔC) and CIITA promoter, luciferase activities were monitored at 24 h after transfection.

To explore whether grass carp IRF1 can activate MHC II expression mediated by CIITA, CIITA and MHC II were detected in GCO cells after transfection of IRF1. As shown in Figure 4A, CIITA, MHC II-α, and MHC II-β were significantly induced. To study whether grass carp CIITA promotes MHC II expression as in mammals, an empty vector and CIITA were respectively transfected into GCO cells. The result indicated that CIITA activated MHC II expression in the absence of IFN-γ and IRF1 (Figure 4B). The Luciferase activity assay also indicated that IRF1 could activate the CIITA promoter (Figure 4C). To verify that IRF1 directly promoted CIITA expression, ChIP assays were performed. The empty vector or IRF1-containing plasmid and CIITA promoter were co-transfected into 293T cells, and the result showed that IRF1 could bind to the CIITA promoter directly (Figure 4D). The promoter sequence of grass carp CIITA gene containing the putative ISREs (−25 to −34 and −45 to −54) was also used to analyze the biological function of IRF1. After mutation of the ISREs, two mutants (Mut 1-pro and Mut 2-pro) of the CIITA promoter were obtained (Figure 4E). The activity of Mut 2-pro was severely suppressed after transfection with IFN-γ or IRF1, which revealed that the second ISRE motif was essential for the binding with IRF1 (Figure 4F) and CIITA promoter activation (Figure 4G). To characterize the involvement of the domains of IRF1 in regulating the CIITA expression, two truncated mutants (Figure 4H), IRF1-ΔN lacking DNA-binding domain (DBD) and IRF1-ΔC containing DBD, were constructed. The results indicated that CIITA expression could not be activated by these two mutants (Figure 4I).

### The Expressions of Grass Carp IRF1 and CIITA After LPS and Poly I:C Stimulation

**Figure 5:**
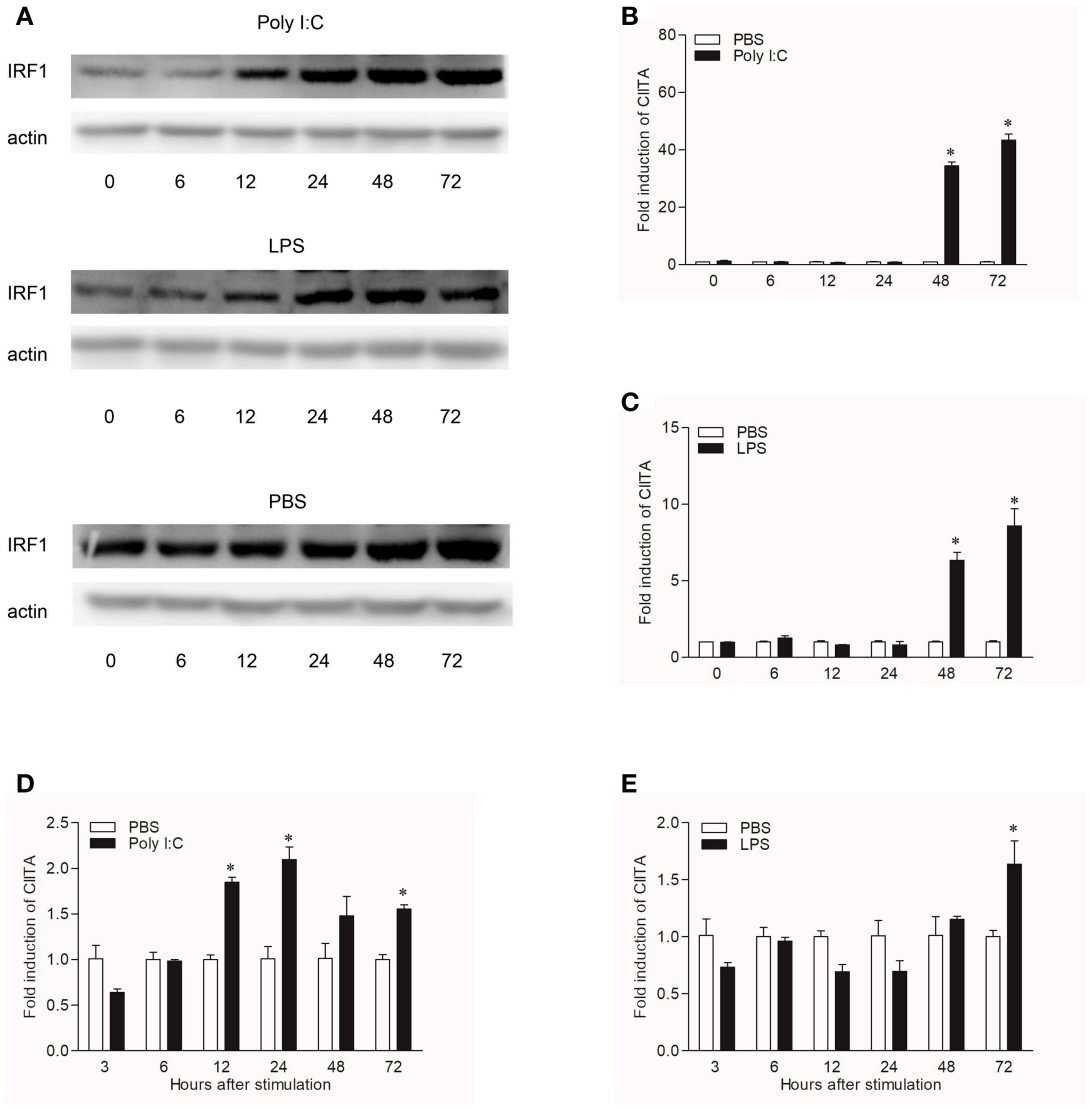
Induction of IRF1 and CIITA by Poly I:C and LPS. **(A)** Grass carp IRF1 expression in CIK cells stimulated with Poly I:C or LPS. Cell samples were harvested at different time points after stimulation and the gene expression were measured through Western blot. **(B,C)** Grass carp CIITA expression in CIK cells stimulated with Poly I:C or LPS. Cells were harvested at different time points after stimulation and fold changes of gene expression were measured through qRT-PCR. The expression level was first normalized to that of β-actin and then a stimulated group divided by that of the time-matched controls. **(D,E)** Grass carp CIITA expression in HKLs stimulated with Poly I:C or LPS. Freshly prepared HKLs were treated with PBS as control or stimulated with Poly I:C or LPS. The mean ± SEM of three fish is shown. Asterisks (^*^) indicate significant differences from control (*p* < 0.05).

In order to explore the expression patterns of CIITA to different stimuli, both CIK cells and grass carp HKLs stimulated with Poly I:C or LPS were collected at different time points. To study the regulation of grass carp IRF1 expression by Poly I:C and LPS, we purified the recombinant IRF1, and the endogenous IRF1 could be detected by the pAb (Figure S4). In CIK cells, IRF1 was measured by Western blot and the result showed that it could be up-regulated at 12 h after Poly I:C or LPS stimulation (Figure 5A), while the transcription level of CIITA could be induced by Poly I:C at 48 h (Figure 5B). The expression profile of CIITA induced by LPS was similar with that induced by Poly I:C (Figure 5C). In HKLs, CIITA expression peaked at 24 h after stimulation with Poly I:C (Figure 5D), while the expression was induced by LPS at 72 h (Figure 5E).

### IRF2 Suppresses MHC II Expression by Competing With IRF1 to Bind to the ISRE of CIITA Promoter

**Figure 6:**
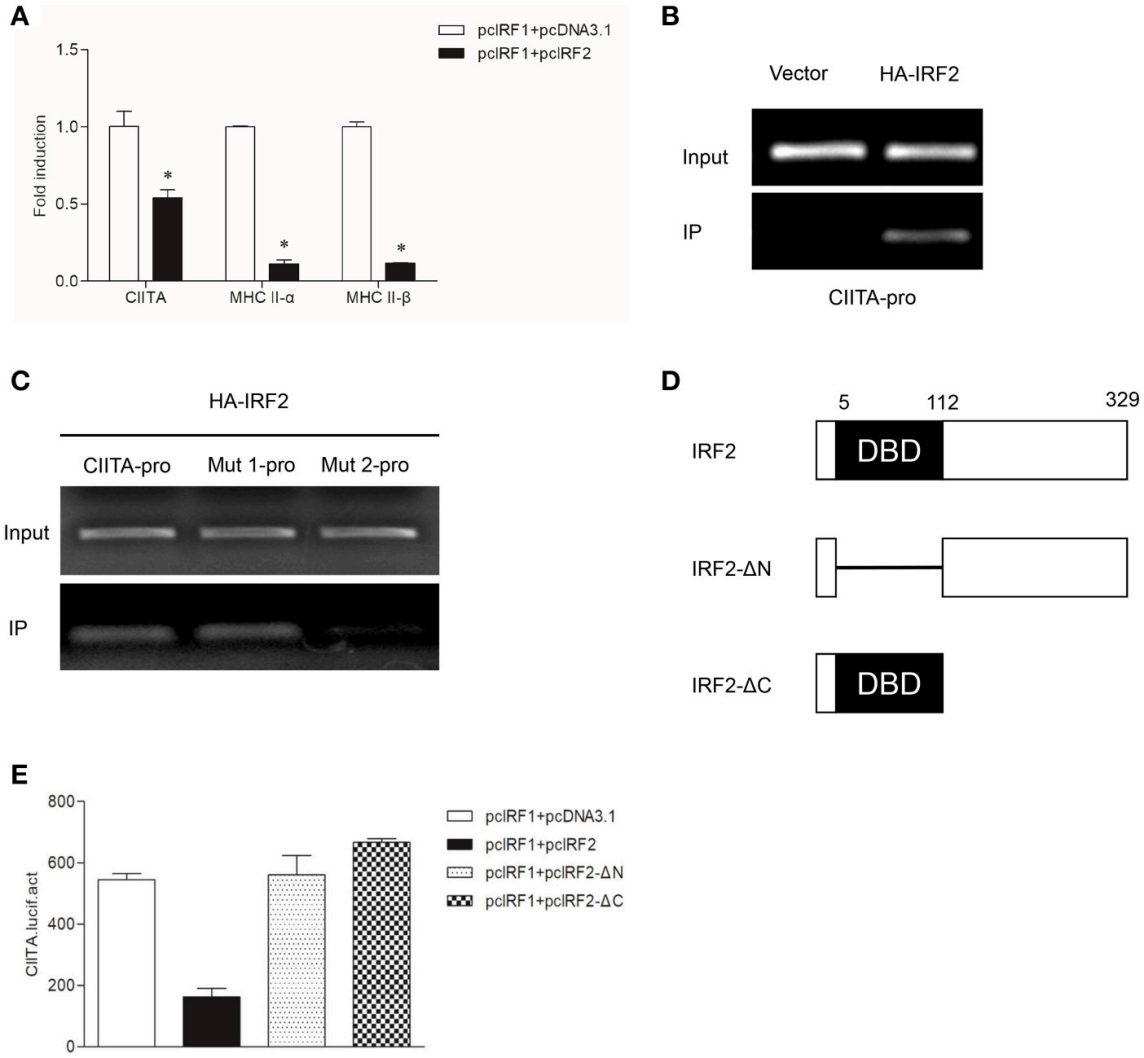
Suppression of CIITA and MHC II expression by IRF2. **(A)** Activation of CIITA and MHC II induced by IRF1 can be suppressed by IRF2. GCO cells seeded in 6-well plate overnight were cotransfected with (pcDNA3.1 or pcIRF2) and pcIRF1. Fold changes of the gene expression were measured through qRT-PCR. Asterisks (^*^) indicate significant differences from control (*p* < 0.05). **(B,C)** The binding of IRF2 with CIITA promoter. The cells seeded in 10-cm dishes were cotransfected with (5 μg PCMV-HA, or HA-IRF2) and (5 μg CIITA-pro, Mut 1-pro, or Mut 2-pro). Cell samples were harvested at 24 h after transfection and cell lysates were immunoprecipitated with anti-HA-Agarose beads. The input was used as a control in semiquantitative PCR to quantify the DNA concentration. **(D)** Schematic representation of wild type IRF2 and its two mutants. **(E)** Effect of the two structural domains of IRF2 on the CIITA promoter. GCO cells were cotransfected with (IRF2, IRF2-ΔN, or IRF2-ΔC) and CIITA promoter, luciferase activities were monitored at 24 h after transfection.

To verify whether grass carp IRF2 can cooperate with IRF1 to activate CIITA expression as that of humans, the empty vector or IRF2 was co-transfected with IRF1 into GCO cells. The results of qRT-PCR showed that IRF2 could inhibit CIITA and MHC II expression (Figure 6A). ChIP assays were conducted as described above, and the results showed IRF2 could directly bind to the CIITA promoter (Figure 6B), and the second ISRE motif was important for the binding (Figure 6C). For further analysis the functional domains of IRF2, two mutants were constructed (Figure 6D). The Dual-luciferase reporter experiment was carried out to detect the function of IRF2-ΔN and IRF2-ΔC, and the results indicated that neither IRF2-ΔN nor IRF2-ΔC could suppress the expression of grass carp CIITA (Figure 6E).

## Discussion

The immune system is highly dynamic and diverse, which enables it to effectively protect an individual from numerous potentially pathogenic encounters. In this process, antigen presentation plays an important role by presenting foreign antigens to induce downstream immune responses. And many studies have shown that MHC II molecules are indispensable in antigen presentation (28, 29). Binding to a foreign antigen, MHC II are then transported to the plasma membrane to present a foreign antigen peptide to CD4^+^ T cells. CIITA, identified as a critical factor required for both the constitutive and inducible expression of MHC II, has been widely evaluated since it was discovered in humans (30). Although we have known that the CIITA gene exists in lower vertebrates (17), no studies have been performed on the characterization, function, and regulatory process of CIITA in grass carp.

In this study, the cDNA and genomic structure of grass carp CIITA were obtained. Analysis from the positions of the first two exons revealed that the cloned CIITA gene was very similar to the human CIITA IV gene, in which the first intron is the shortest one among the four CIITA genes. The predicted protein domain analysis of grass carp CIITA indicated that it contains three GTP-binding motifs, which might endow it with GTP-binding activity. In mammals, CIITA can bind to GTP, and mutation of any of the three GTP-binding motifs will abrogate the GTP binding activity of CIITA and lead to CIITA failing to localize to the nucleus (31). The NLS (KKRKK) of simian virus 40 (SV40), recognized by the importins, has become the classic model for the nuclear import of proteins (32). In bare lymphocyte syndrome (BLS) patient, a CIITA lacking 24-amino acid in the C terminus was identified, and the 5-amino-acid motif within the lacking sequence is very similar to the NLS of SV40 (30). As a nuclear transcription factor, grass carp CIITA also contains a similar NLS (KKRK), which might be essential for its nuclear localization. In addition, LRR regions of CIITA have been shown to mediate protein-protein interaction and self-association events (33), and the regions were also characterized in grass carp CIITA. The protein blast of CIITA indicated that grass carp CIITA has five LRR regions, while human CIITA has only three LRR regions.

Expression analysis showed that grass carp CIITA was expressed in all tissues. As compared with the non-immune tissues, CIITA was significantly expressed more in the kidney, spleen, and blood, suggesting a potentially vital role of this gene in the systemic immune response of grass carp. The medium expression of CIITA was detected in the gill and gut, implying that CIITA may also play an important role in mucosal immunity. After stimulation with Poly I:C and LPS, a high expression of CIITA in HKLs were respectively detected at 24 h and 72 h, suggesting that CIITA showed different expression patterns to different antigens. In CIK cells, IRF1 were also induced to regulate the expression of CIITA at 12 h after Poly I:C or LPS stimulation, and CIITA showed higher level at 48 h after stimulation. These data suggest that CIITA is important in the immune system of bony fish to defend against the invasion of viral or bacterial pathogens.

Interestingly, reports have shown there are two IFN-γ-like genes, later named IFN-γ and IFN-γ rel, existing in bony fish (18). These two genes may play roles in the antigen presentation signaling pathway mediated by CIITA. Regulation of MHC II expression induced by IFN-γ and IFN-γ rel were then explored in grass carp cell lines. The results showed that only IFN-γ could induce the expression of STAT1a, STAT1b, IRF1, CIITA, and MHC II, suggesting a conserved function of homologous genes in different species. As for IFN-γ rel, it could not induce the expression of genes related to antigen presentation, indicating IFN-γ rel may play other roles in the immune process. In the absence of IFN-γ, the MHC II expression could be induced by IRF1 or CIITA, indicating these two molecules packed in nanoparticles can be used as an antigen presenting activator in fish.

**Figure 7:**
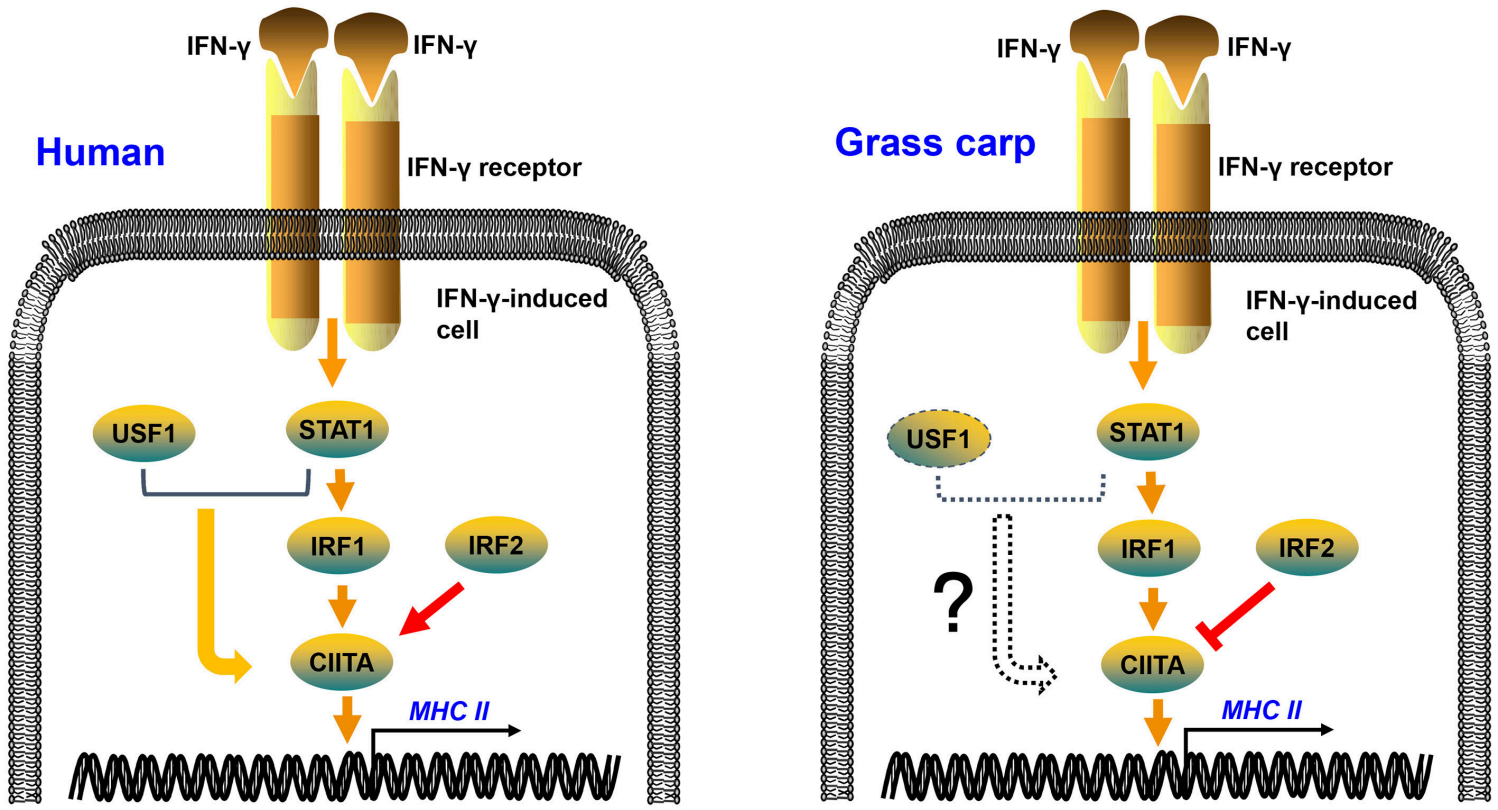
Regulation mechanisms of IFN-γ-induced MHC II expression in human and grass carp. After stimulation with IFN-γ, STAT1, IRF1, CIITA, and MHC II can be induced in human and grass carp. IRF2 and IRF1 cooperatively induce the activation of CIITA in human, while IRF2 binds to the promoter of CIITA and suppresses the expression of CIITA in grass carp. STAT1 and USF1 can cooperatively activate the expression of CIITA in human, but this mechanism may not exist in grass carp because of the absence of STAT1 and USF1 binding sites in the CIITA promoter.

In mammals, IRF1 and IRF2 can simultaneously occupy the ISRE motif of the type IV CIITA promoter, and then cooperatively induce the activation of CIITA (34, 35). As in mammals, grass carp IRF1 and IRF2 could bind to the ISRE motif of the CIITA promoter; but IRF2 played an antagonistic role to IRF1 in transcriptional regulation of CIITA and MHC II in grass carp (Figure 7). This may be a difference between fish and higher vertebrates in the regulation of antigen presentation. The two mutants of IRF1 could not activate the CIITA promoter, suggesting that both the N and C regions of IRF1 are essential for the activation of CIITA. The two mutants of IRF2 could not suppress the expression of CIITA, indicating that the complete structural domain is essential to the role of IRF2. ChIP assays demonstrated that both IRF1 and IRF2 could bind to the second ISRE motif of the CIITA promoter, implying that IRF2 may competitively bind to this motif, thereby interrupting activation of the CIITA gene induced by IRF1. As for another IRF1 family member, IRF11 could not be induced by IFN-γ and IFN-γ rel, and had no active function in the expression of grass carp CIITA (Figure S5). In fact, research has mainly focused on the molecular characterization and expression analysis of IRF11 (36, 37), and its function has not been explored thoroughly in bony fish. In addition, Phosphorylated STAT1 and upstream transcription factor 1 (USF-1), which, respectively binds to GAS and E box, can also cooperatively induce the activation of CIITA in mammals (38). The putative GAS and E box motifs have not been found in grass carp CIITA promoter, which suggests that the unique mechanism in human may emerge during the evolution (Figure 7). Certainly, this hypothesis requires further verification.

The host has a variety of defense mechanisms, including both innate and acquired immunity, to prevent invasions of viruses or bacteria. While a successful vertebrate pathogen must overcome or alter many normally effective host defense mechanisms to avoid being cleared (39). In mammals, the mechanisms of pathogen evading antigen presentation have been extensively studied (40–42). In bony fish, the research mainly focused on the escape of pathogens from innate immune signal pathways, especially the interferon-related signal pathways (43, 44). In grass carp, RNA-seq profiles from tissues indicated that MHC II was downregulated after grass carp reovirus (GCRV) infection, and the researcher speculated that there might be a mechanism inhibiting the activation of T cells (45). Perhaps due to the incomplete knowledge of fish antigen presentation mechanisms, the study on aquatic animal pathogens evading host antigen presentation has not been reported. In this study, the IFN-γ-induced MHC II expression signaling pathway has been elucidated, laying a foundation for further studies on the mechanisms how pathogens escape from antigen presentation in grass carp.

## Ethics Statement

All operations of fish were approved by the Committee on the Ethics of Animal Experiments of the Institute of Hydrobiology, Chinese Academy of Sciences.

## Author Contributions

X-BL performed most of the experiments, analyzed the data, and wrote the manuscript. Z-XW helped with the partial plasmid construction. S-BL and X-YZ helped with the preparation of experimental samples. L-FL and SL helped with experimental operations. D-DC helped with the preparation of antiserum. PN helped with experiment design. Y-AZ designed the research and revised the manuscript.

### Conflict of Interest Statement

The authors declare that the research was conducted in the absence of any commercial or financial relationships that could be construed as a potential conflict of interest.

## Abbreviations

APC: antigen-presenting cell
CIITA: MHC class II transactivator
DC: dendritic cell
GBD: GTP-binding domain
IRF: interferon regulatory factor
ISRE: interferon stimulation response element
LRR: leucine-rich repeat
MHC II: major histocompatibility complex II
NLS: nuclear localization sequence
STAT1: signal transducer and activator of transcription 1.

## References

[1] Roche PA Furuta K. The ins and outs of MHC class II-mediated antigen processing and presentation. Nat Rev Immunol. (2015) 15:203–16. 10.1038/nri3818 25720354 PMC6314495

[2] Starr TK Jameson SC Hogquist KA. Positive and negative selection of T cells. Annu Rev Immunol. (2003) 21:139–76. 10.1146/annurev.immunol.21.120601.141107 12414722

[3] Londhe P Davie JK. Gamma interferon modulates myogenesis through the major histocompatibility complex class II transactivator, CIITA. Mol Cell Biol. (2011) 31:2854–66. 10.1128/MCB.05397-11 21576360 PMC3133399

[4] Romieu MR François M Boivin MN Stagg J Galipeau J Regulation of MHC class II expression and antigen processing in murine and human mesenchymal stromal cells by IFN-γ, TGF-β, and cell density. J Immunol. (2007) 179:1549–58. 10.4049/jimmunol.179.3.1549 17641021

[5] Geppert TD Lipsky PE. Antigen presentation by interferon-gamma-treated endothelial cells and fibroblasts: differential ability to function as antigen-presenting cells despite comparable Ia expression. J Immunol. (1985) 135:3750–62. 3934267

[6] Leibund LS Waldburger JM Krawczyk M Otten LA Suter T Fontana A Mini-review: specificity and expression of CIITA, the master regulator of MHC class II genes. Eur J Immunol. (2004) 34:1513–25. 10.1002/eji.200424964 15162420

[7] Rohn WM Lee YJ Benveniste EN. Regulation of class II MHC expression. Crit Rev Immunol. (1996) 16:311–30. 10.1615/CritRevImmunol.v16.i3.40 8922901

[8] Pai RK Askew D Boom WH Harding CV. Regulation of class II MHC expression in APCs: roles of types I, III, and IV class II transactivator. J Immunol. (2002) 169:1326–33. 10.4049/jimmunol.169.3.1326 12133955

[9] Ting JP Trowsdale J. Genetic control of MHC class II expression. Cell. (2002) 109:S21–33. 10.1016/S0092-8674(02)00696-7 11983150

[10] Jabrane-Ferrat N Nekrep N Tosi G Esserman LJ Peterlin BM. Major histocompatibility complex class II transcriptional platform: assembly of nuclear factor Y and regulatory factor X (RFX) on DNA requires RFX5 dimers. Mol Cell Biol. (2002) 22:5616–25. 10.1128/mcb.22.15.5616-5625.2002 12101253 PMC133954

[11] Wright KL Chin KC Linhoff M Skinner C Brown JA Boss JM. CIITA stimulation of transcription factor binding to major histocompatibility complex class II and associated promoters *in vivo*. Proc Natl Acad Sci USA. (1998) 95:6267–72. 9600954 10.1073/pnas.95.11.6267 PMC27653

[12] Muhlethaler-Mottet A Otten LA Steimle V Mach B. Expression of MHC class II molecules in different cellular and functional compartments is controlled by differential usage of multiple promoters of the transactivator CIITA. EMBO J. (1997) 16:2851–60. 10.1093/emboj/16.10.2851 9184229 PMC1169893

[13] Reith W LeibundGut-Landmann S Waldburger JM. Regulation of MHC class II gene expression by the class II transactivator. Nat Rev Immunol. (2005) 5:793–806. 10.1038/nri1708 16200082

[14] Dong Y Rohn WM Benveniste EN. IFN-γ regulation of the type IV class II transactivator promoter in astrocytes. J Immunol. (1999) 162:4731–9. 10202014

[15] Ramana CV Gil MP Schreiber RD Stark GR. Stat1-dependent and -independent pathways in IFN-γ-dependent signaling. Trends Immunol. (2002) 23:96–101. 10.1016/S1471-4906(01)02118-4 11929133

[16] Morris AC Beresford GW Mooney MR Boss JM. Kinetics of a gamma interferon response: expression and assembly of CIITA promoter IV and inhibition by methylation. Mol Cell Biol. (2002) 22:4781–91. 10.1128/MCB.22.13.4781-4791.2002 12052885 PMC133907

[17] Liu Y Meng Y Wang Q Sha Z. Class II, major histocompatibility complex, transactivator (CIITA) in channel catfish: identification and expression patterns responding to different pathogens. Mol Biol Rep. (2012) 39:11041–50. 10.1007/s11033-012-2007-z 23073771

[18] Igawa D Sakai M Savan R. An unexpected discovery of two interferon gamma-like genes along with interleukin (IL)-22 and−26 from teleost: IL-22 and−26 genes have been described for the first time outside mammals. Mol Immunol. (2006) 43:999–1009. 10.1016/j.molimm.2005.05.009 16005068

[19] Milev MI Long S Wilson M Bengten E Miller NW Chinchar VG Identification and expression analysis of interferon gamma genes in channel catfish. Immunogenetics. (2006) 58:70–80. 10.1007/s00251-006-0081-x 16467986

[20] Stolte EH Savelkoul HFJ Wiegertjes G Flik G Lidy Verburg-van Kemenade BM. Differential expression of two interferon-γ genes in common carp (*Cyprinus carpio L*.). Dev Comp Immunol. (2008) 32:1467–81. 10.1016/j.dci.2008.06.012 18657572

[21] Chen WQ Xu QQ Chang MX Zou J Secombes CJ Peng KM. Molecular characterization and expression analysis of the IFN-gamma related gene (IFN-γrel) in grass carp *Ctenopharyngodon idella*. Vet Immunol Immunopathol. (2010) 134:199–207. 10.1016/j.vetimm.2009.09.007 19800136

[22] Shibasaki Y Yabu T Araki K Mano N Shiba H Moritomo T. Peculiar monomeric interferon gammas, IFNγrel 1 and IFNγrel 2, in ginbuna crucian carp. FEBS J. (2013) 281:1046–56. 10.1111/febs.12666 24373358

[23] Stein C Caccamo M Laird G Leptin M. Conservation and divergence of gene families encoding components of innate immune response systems in zebrafish. Genome Biol. (2007) 8:R251. 10.1186/gb-2007-8-11-r251 18039395 PMC2258186

[24] Shu C Sun Y Xu T. Molecular characterization of three IRF1 subfamily members reveals evolutionary significance of IRF11 in miiuy croaker. Dev Comp Immunol. (2015) 53:385–91. 10.1016/j.dci.2015.07.009 26187301

[25] Zhang YA Hikima J Li J LaPatra SE Luo YP Sunyer JO. Conservation of structural and functional features in a primordial CD80/86 molecule from rainbow trout (*Oncorhynchus mykiss*), a primitive teleost fish. J Immunol. (2009) 183:83–96. 10.4049/jimmunol.0900605 19535623

[26] Wang TH Diaz-Rosales P Costa MM Campbell S Snow M Collet B. Functional characterization of a nonmammalian IL-21: rainbow trout *Oncorhynchus mykiss* IL-21 upregulates the expression of the Th cell signature cytokines IFN-γ, IL-10, and IL-22. J Immunol. (2011) 186:708–21. 10.4049/jimmunol.1001203 21160047

[27] Li S Lu LF Feng H Wu N Chen DD Zhang YB. IFN regulatory factor 10 is a negative regulator of the IFN responses in fish. J Immunol. (2014) 193:1100–9. 10.4049/jimmunol.1400253 24958903

[28] Broeke T Wubbolts R Stoorvogel W. MHC Class II Antigen presentation by dendritic cells regulated through endosomal sorting. CSH Perspect Biol. (2013) 5:a016873. 10.1101/cshperspect.a016873 24296169 PMC3839614

[29] Watts C. The exogenous pathway for antigen presentation on major histocompatibility complex class II and CD1 molecules. Nat Immunol. (2004) 5:685–92. 10.1038/ni1088 15224094

[30] Steimle V Otten LA Zufferey M Mach B. Complementation cloning of an MHC class II transactivator mutated in hereditary MHC class II deficiency (or bare lymphocyte syndrome). Cell. (1993) 75:135–46. 10.1016/0092-8674(93)90685-J 8402893

[31] Harton JA Cressman DE Chin KC Der CJ Ting JP. GTP binding by class II transactivator: role in nuclear import. Science. (1999) 285:1402. 10.1126/science.285.5432.1402 10464099

[32] Harton JA Ting JP. Class II transactivator: mastering the art of major histocompatibility complex expression. Mol Cell Biol. (2000) 20:6185–94. 10.1128/MCB.20.17.6185-6194.2000 10938095 PMC86093

[33] Linhoff MW Harton JA Cressman DE Martin BK Ting JP. Two distinct domains within CIITA mediate self-association: involvement of the GTP-binding and leucine-rich repeat domains. Mol Cell Biol. (2001) 21:3001–11. 10.1128/MCB.21.9.3001-3011.2001 11287606 PMC86929

[34] Xi H Eason DD Ghosh D Dovhey S Wright KL Blanck G. Co-occupancy of the interferon regulatory element of the class II transactivator (CIITA) Type IV promoter by interferon regulatory factors 1 and 2. Oncogene. (1999) 18:5889–903. 10.1038/sj.onc.1202969 10557076

[35] Xi H Blanck G. The IRF-2 DNA binding domain facilitates the activation of the class II transactivator (CIITA) type IV promoter by IRF-1. Mol Immunol. (2003) 39:677–84. 10.1016/S0161-5890(02)00214-6 12493643

[36] Mao F Lin Y Zhou Y He Z Li J Zhang Y. Structural and functional analysis of interferon regulatory factors (IRFs) reveals a novel regulatory model in an invertebrate, *Crassostrea gigas*. Dev Comp Immunol. (2018) 89:14–22. 10.1016/j.dci.2018.07.027 30077552

[37] Zhang J Li YX Hu YH. Molecular characterization and expression analysis of eleven interferon regulatory factors in half-smooth tongue sole, *Cynoglossus semilaevis*. Fish Shellfish Immunol. (2015) 44:272–82. 10.1016/j.fsi.2015.02.033 25731919

[38] Muhlethaler-Mottet A Di Berardino W Otten LA Mach B. Activation of the MHC Class II transactivator CIITA by interferon-γ requires cooperative interaction between Stat1 and USF-1. Immunity. (1998) 8:157–66. 10.1016/S1074-7613(00)80468-9 9491997

[39] Finlay BB McFadden G. Anti-immunology: evasion of the host immune system by bacterial and viral pathogens. Cell. (2006) 124:767–82. 10.1016/j.cell.2006.01.034 16497587

[40] Li D Qian L Chen C Shi M Yu M Hu M. Down-regulation of MHC class II expression through inhibition of CIITA transcription by lytic transactivator Zta during Epstein-Barr virus reactivation. J Immunol. (2009) 182:1799–809. 10.4049/jimmunol.0802686 19201831

[41] Abendroth A Slobedman B Lee E Mellins E Wallace M Arvin AM. Modulation of major histocompatibility class II protein expression by varicella-zoster virus. J Virol. (2000) 74:1900–7. 10.1128/JVI.74.4.1900-1907.2000 10644363 PMC111668

[42] Cai Q Banerjee S Cervini A Lu J Hislop AD Dzeng R. IRF-4-mediated CIITA transcription is blocked by KSHV encoded LANA to inhibit MHC II presentation. PLoS Pathog. (2013) 9:e1003751. 10.1371/journal.ppat.1003751 24204280 PMC3814934

[43] Lu LF Li S Wang ZX Du SQ Chen DD Nie P Grass carp reovirus VP41 targets fish MITA to abrogate the IFN response. J Virol. (2017) 91: e00390–17. 10.1128/JVI.00390-17 28446676 PMC5487562

[44] Lu LF Li S Lu XB LaPatra SE Zhang N Zhang XJ Spring viremia of carp virus N protein suppresses fish IFNϕ1 production by targeting the mitochondrial antiviral signaling protein. J Immunol. (2016) 196:3744–53. 10.4049/jimmunol.1502038 26994222

[45] Shi M Huang R Du F Pei Y Liao L Zhu Z. RNA-seq profiles from grass carp tissues after reovirus (GCRV) infection based on singular and modular enrichment analyses. Mol Immunol. (2014) 61:44–53. 10.1016/j.molimm.2014.05.004 24865419

