# Supplementary material for: Interferon Regulatory Factors 1 and 2 Play Different Roles in MHC II Expression Mediated by CIITA in Grass Carp, Ctenopharyngodon idella
Source: Front Immunol. 2019 May 22;10:1106. doi: 10.3389/fimmu.2019.01106 (PMC6540827; doi:10.3389/fimmu.2019.01106)
Supplement: Supplementary file 1 [file Data_Sheet_1.PDF]

Supplementary Material

Supplementary Table

Table S1. Primers used in this study

Primer	Sequence (5’-3’)	Purpose
CIITA-dp-F	AGYGATTCAGAGCGAAAGAAAGC	Gene cloning
CIITA-dp-R	CTCGTAMACACARTGAAACARTTC	
IRF11-dp-F	CCTGGCTGGAGGAACAGATC	
IRF11-dp-R	GCGTTGTGGCCTTTCTTGATG	
CIITA-5’-F1	CTGTAGTTTGATTGTGTGAGGTCT	5’-RACE
CIITA-5’-F2	TCCGAGGAGAGCGACAGATTGT	
IRF11-5’-F1	GCATGTTTCCAGGGAATCTGG	
IRF11-5’-F2	CAGCCACACGACTCCAGGAT	
CIITA-3’-R1	TGGCAAACAGCATAGTCCAGCAG	3’-RACE
CIITA-3’-R1	AGAGTCAGCATCCTGGACAGCA	
IRF11-3’-R1	AAGCAAACTTCCGATGCGCAC	
IRF11-3’-R1	CGGACGTCAAAGAGCTTCAAG	
C Ⅱ TA-Full-F	ATGCTGAATTTCAAGATATTGTCTC	Sequence verification
C Ⅱ TA-Full-R	TCAAAGGGATCTTATTCTTGAATC	
IRF11-Full-F	ATGCATCAGGGACGCCTACG	
IRF11-Full-R	TTAGCCTGATGAGGTATTGTGAA	
pc-IFN-γ-F	GGGGTACCATGGATTCTTGGCTCAACATGAT	Vector construction
pc-IFN-γ-R	ATTTGCGGCCGCTCATTGAACCTTTTTGTGTTTTTCT	
pc-IFN-γ rel-F	GGGGTACCATGATTGCACAACACATGATGG	
pc-IFN-γ rel-R	ATTTGCGGCCGCCTAAGACTTCTGCCTCTTGATG	
Pc-IRF1-F	GGGGTACCATGCCTGTGTCCAGAATGCGCA	
Pc-IRF1-F	CCGCTCGAGTCAGAGAGGACACATGGTCG	
Pc-IRF1-ΔC-F	CGGAATTCGGATGCCTGTGTCCAGAATGCGCA	
Pc-IRF1-ΔC-R	CCGCTCGAGTCACAGCATGCGGTAGACTCG	
Pc-IRF1-ΔN-F	CGGAATTCGGATGCCTGTGTCCAGAATGCCCGCCGTGAGCA AGAAGA	
Pc-IRF1-ΔN-R	CCGCTCGAGTCAGAGAGGACACATGGTCG	
Myc-IRF1-F	CGGAATTCGGATGCCTGTGTCCAGAATGCGCA	
Myc-IRF1-R	CCGCTCGAGTCAGAGAGGACACATGGTCG	
Pc-IRF2-F	CGGAATTCATGCCGGTAGAGAGAATGCG	
Pc-IRF2-R	CCGCTCGAGTCAGCAGCTCTTGACGGAG	
Pc-IRF2-ΔC-F	CGGAATTCATGCCGGTAGAGAGAATGCGT	
Pc-IRF2-ΔC-R	CCGCTCGAGTCACGCTGACAGCATCTTGTACAC	

Pc-IRF2-ΔN-F	CGGAATTCATGCCGGTAGAGAGAATGCGTTCTGAGAAGCAT TCAAAAGGA	
Pc-IRF2-ΔN-R	CCGCTCGAGTCAGCAGCTCTTGACGGAGG	
HA-IRF2-F	CGGAATTCGGATGCCGGTAGAGAGAATGCG	
HA-IRF2-R	CCGCTCGAGTCAGCAGCTCTTGACGGAG	
Pc-IRF11-F	CGGAATTCATGCATCAGGGACGCCTACG	
Pc-IRF11-R	CCGCTCGAGTTAGCCTGATGAGGTATTGTGAA	
qSTAT1a-F	TGTCAGAGAATCAAACCAGCAG	qRT-PCR
qSTAT1a-R	CTTGAAGTCGTATTCGTCTTGC	
qSTAT1b-F	ATGAGCACTACAGCCGTCTCA	
qSTAT1b-R	TCATTCAGAGTATTAGCTATGATC	
qIRF1-F	CTATCATTGAGATTTCACGGCAA	
qIRF1-R	AAGCTCCTCTCCTGAACTTACA	
qIRF11-F	AAGCAGTGCTGAAGATTGTGGA	
qIRF11-R	GTAACCTGAAGATTCCATCGTC	
qCIITA-F	TGGAGATGCCGGTGTGGAGA	
qCIITA-R	CAGGTGTTCTGCTCCAATGTC	
qMHC II-α-F	AGAGGGAGACATTTACAGTTGC	
qMHC II-α-R	CAGCGACTCCCAGCAGACC	
qMHC II-β-F	ACTGGTACTACCAGATTCACTC	
qMHC II-β-R	CCACACAGGAGATCTTCTCTC	
ChIP-CIITA-F	TGCTAAGCCACCAAGGAAGGT	ChIP
ChIP-CIITA-R	CACGACGTGTCCTCAGATTAC	

Supplementary Figures

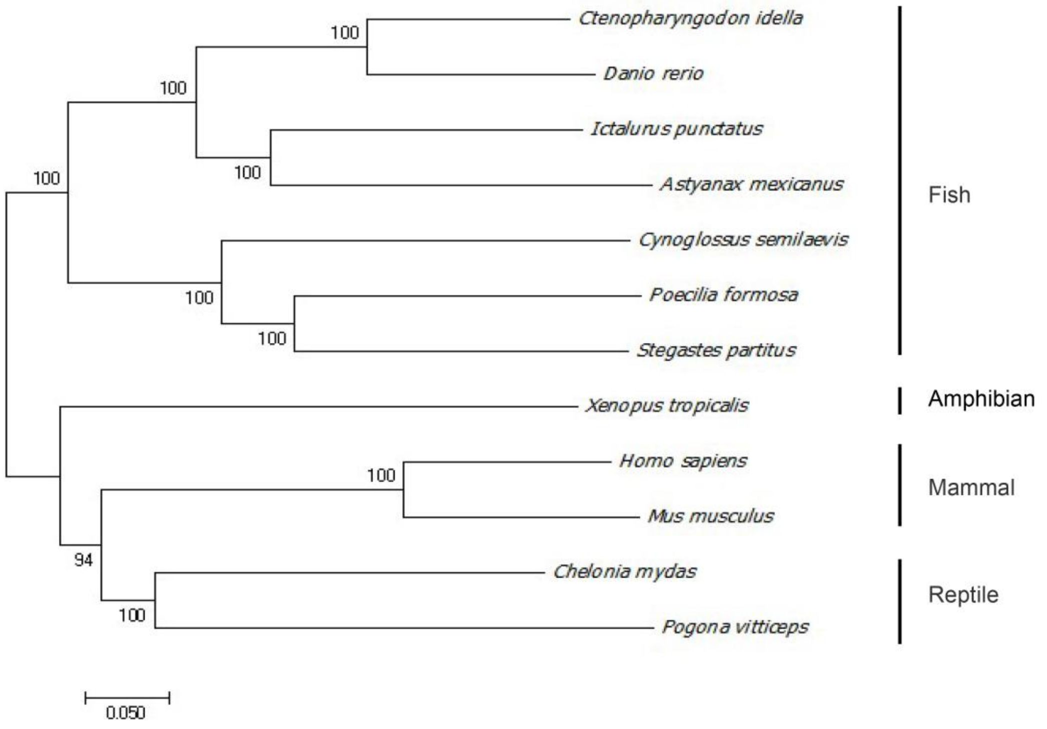

Figure S1. Phylogenetic trees of vertebrate CIITA constructed by neighbor-joining method based on the amino acid alignment. The accession numbers of CIITA included in the phylogenetic tree are as follows: *Homo sapiens*, NP_001273331; *Mus musculus*, NP_001230689; *Pogona vitticeps*, XP_020664086; *Chelonia mydas*, XP_007058212; *Xenopus tropicalis*, XP_017952759; *Stegastes partitus*, XP_008290065; *Poecilia formosa*, XP_007565831; *Cynoglossus semilaevis*, XP_008328336; *Astyanax mexicanus*, XP_007245634.1; *Ictalurus punctatus*, AFL70283; *Danio rerio*, XP_009297712; *Ctenopharyngodon idella*, AXY05349.

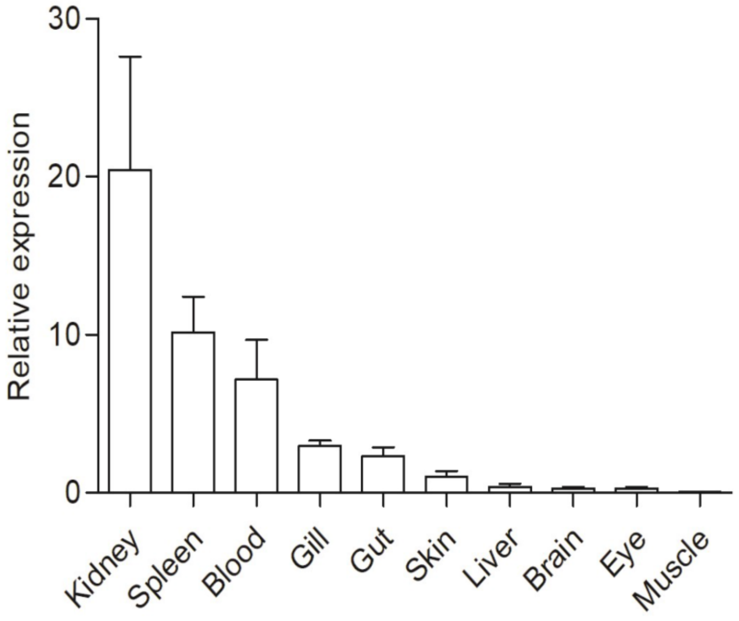

Figure S2. Tissue expression of CIITA in grass carp. The tissues were collected from three individuals, including head kidney, spleen, blood, gill, gut, skin, liver, brain, eye, and muscle. The transcripts of CIITA were measured by qRT-PCR. CIITA expression in skin was set as a baseline from which all other values were normalized.

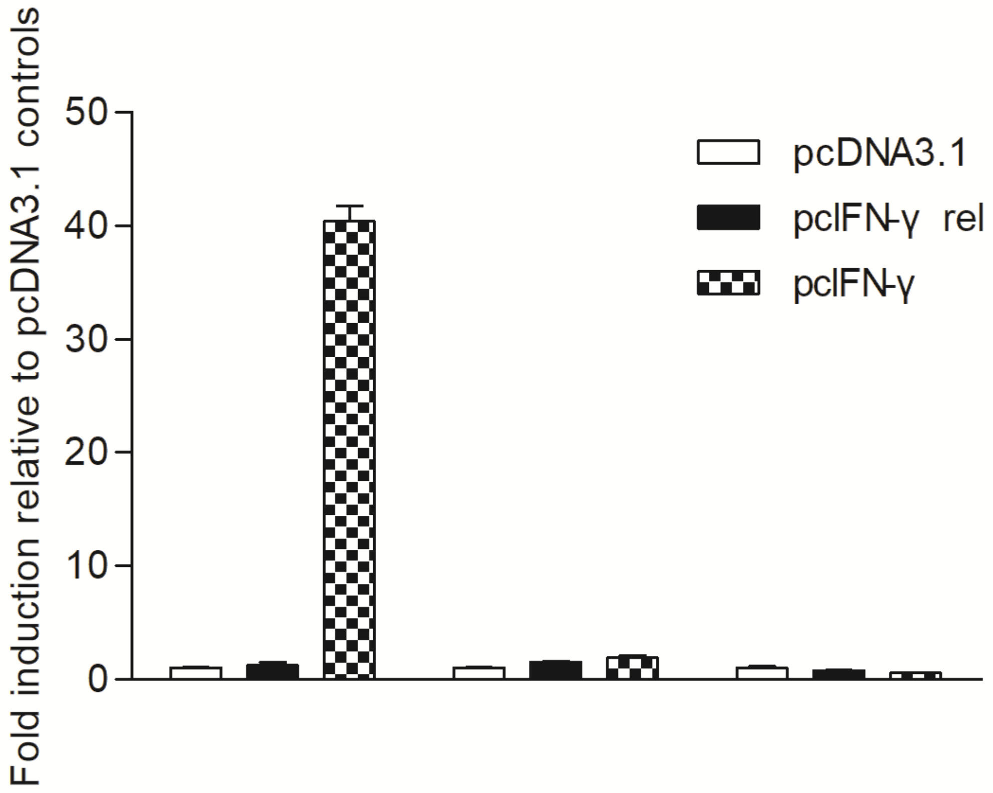

Figure S3. Activation of IRF1, IRF2 and IRF11 by IFN-γ and IFN-γ rel. In GCO cells, fold changes of gene expression were measured at 24 h after transfection of pcDNA3.1, IFN-γ or IFN-γ-rel plasmids. Then total RNAs were extracted to examine the mRNA levels of IRF1, IRF2, and IRF11 through qRT-PCR.

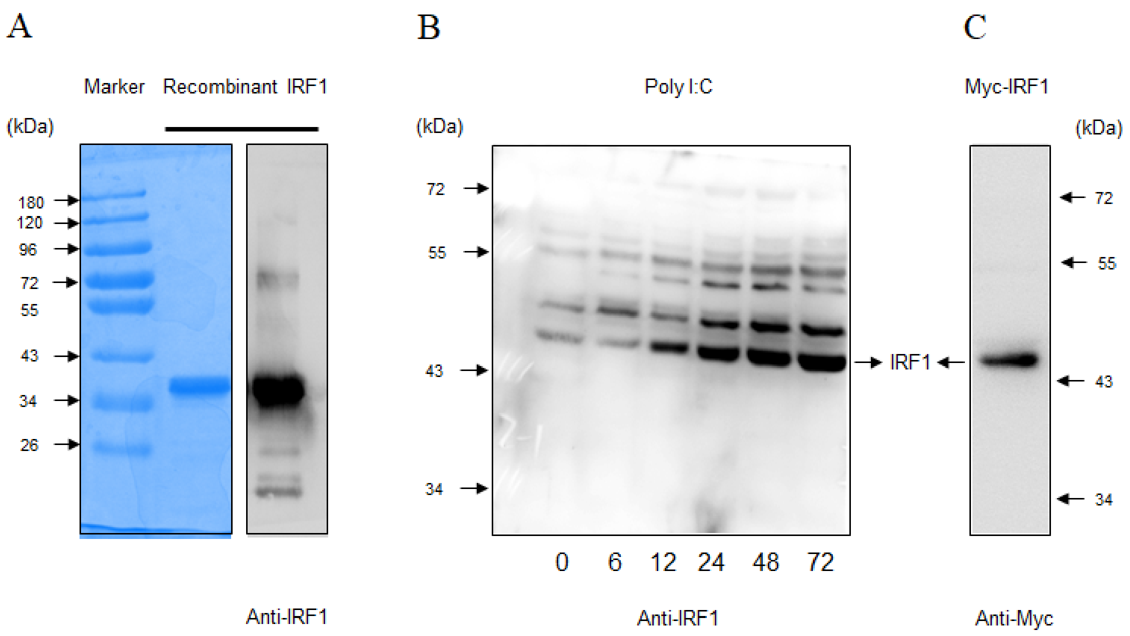

Figure S4. Purification of recombinant IRF1 and confirmation of polyclonal antibody. **(A)** Recombinant IRF1 was analyzed by SDS-PAGE under reducing conditions and stained with Coomassie blue R250. Recombinant IRF1 was also analyzed by Western blot with the polyclonal antibody. **(B)** Endogenous IRF1 expression in CIK cells induced by Poly I:C at different time points was detected with the polyclonal antibody. **(C)** Detection of eukaryotic expression of IRF1 containing an N-terminal Myc-tag. 293T cells seeded in 6-well plate were transfected with 2 μg Myc-IRF1. Cell sample was harvested at 24 h after transfection and cell lysate was detected by Western blot with anti-Myc antibody.

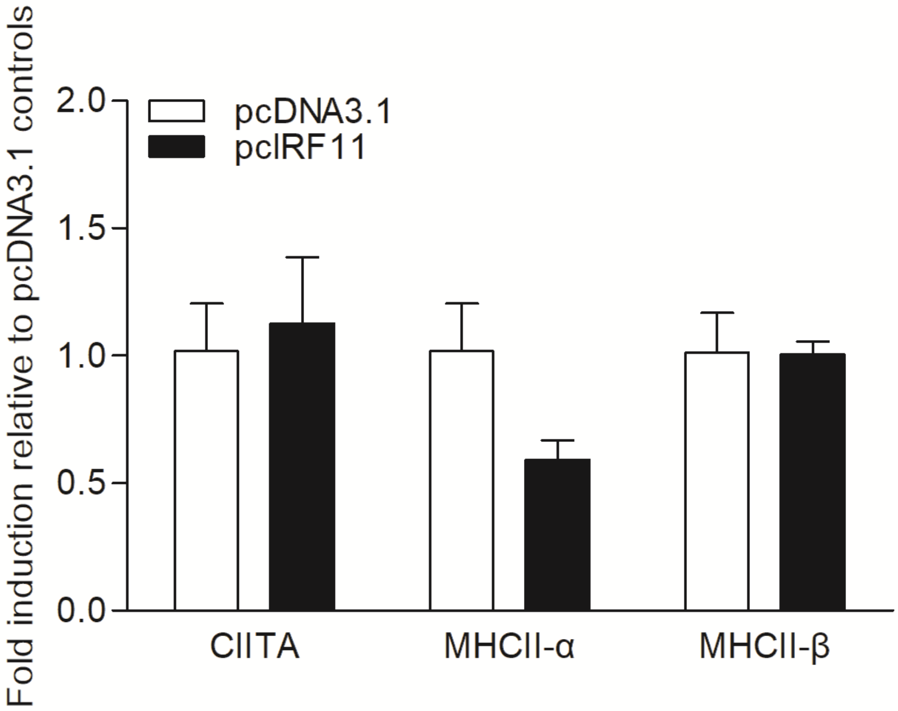

Figure S5. Activation of CIITA and MHC II by IRF11. In GCO cells, fold changes of gene expression were measured at 48 h after transfection of pcDNA3.1 or IRF11. Total RNAs were extracted to examine the mRNA levels of CIITA and MHC II through qRT-PCR.
